# Anthropomorphism in Human–Animal Interactions: A Pragmatist View

**DOI:** 10.3389/fpsyg.2018.02590

**Published:** 2018-12-18

**Authors:** Véronique Servais

**Affiliations:** Laboratory of Social and Cultural Anthropology, Faculty of Social Sciences, University of Liège, Liège, Belgium

**Keywords:** anthropomorphism, human–animal interaction, animism, pragmatism, relationality, expressive gesture, direct perception

## Abstract

This paper explores anthropomorphism in human–animal interactions from the theoretical perspectives of pragmatism and anthropology of human–animal communication. Its aim is to challenge the conception of anthropomorphism as the attribution/inference of human properties to a non-human animal – particularly as a special case of the theory of mind. The author’s goal is to articulate a plausible an alternative conception of anthropomorphism as a situated direct perception of human properties by someone who is engaged in a given situation and sensitive to what the animal is doing to them. Rooted in pragmatist theory as well as in contemporary anthropological studies, this paper offers an original perspective for in depth ethnographic and empirical studies of anthropomorphism-in-situation. Such studies could bring new insights in the study of how ordinary people make sense of animal behaviors in real-life situations.

## Introduction

How do people manage to make sense of animals? One answer, provided by the biologist and anthropologist Bateson (Bateson), is that people make sense of plants or animals when they can perceive the “pattern which connect” them to that animal or plant. As a form of poetic introduction to the problem of anthropomorphism, I would like to cite an excerpt from a poem by William Wordsworth already used by Bateson in a paper about aesthetics entitled “The creature and its creation.” In this poem Wordsworth mocks a man in these words:

‘A primrose by a river’s brim
A yellow primrose was to him
And it was nothing more’

Because the primrose is “just” a yellow thing over there, this man is unable to relate to the flower. In contrast with this man, stands the poet, to whom, according to Bateson, the primrose can be something more: a self-reflexive recognition. “The primrose resembles a poem and both poem and primrose resemble the poet.” (Bateson, 1974–1991, p. 269). Something of the poet is perceived to be present “in” the primrose itself. In the introductory section of Mind and Nature (Bateson, 1979), Bateson also evokes this poem, arguing that this recognition allows the primrose to become *relevant* to the poet, because through this recognition, the poet discovers that he is part of the same story as the primrose. Thus, according to Bateson, to make sense of something is to be able to share a story with it, and this is how people make their environment – both human and non-human– *relevant*. “I would assume that any A is relevant to any B if both A and B are parts or components of the same ‘story’ (Bateson, 1979, p. 13) – and for Bateson, being a part of biological evolution is sharing a story.

At first sight, the aesthetic experience of the poet has nothing in common with anthropomorphism, if anthropomorphism is defined as the conscious “attribution” or “inference” of human characteristics to a non-human being. The poet’s experience seems to be completely different: it has to do with the *direct perception* of some human – yet indeterminate – qualities in the flower.

Still, the story of the primrose indicates the direction of the discussion that will follow. The main argument is that in everyday life, animal mental qualities are not so much inferred as they are recognized, or directly perceived, by a human being who is engaged in a specific interaction. This point of view is defended by Gallagher (2008) in the context of human social cognition and by Morris (2017) in the context of human-animal interaction. The approach advocated here is in line with the “embodiment approaches” that Morris (2017) identifies as promising alternatives to the theory-of-mind approaches of animal minding. Morris criticizes the theory-of-mind approaches because they all assume that “there must be some cognitive process or mechanism that is operating to allow people to bridge the gap between observable behavior and mind” (Morris, 2017, p. 2). On the contrary, says he, the essence of embodiment approaches is that “mind is embodied in behavior” or that “mind is directly available in behavior” (ibid., p. 3).

Accordingly, the aim of this paper is to provide a convincing theoretical framework to support such a claim. It draws mainly on the pragmatist perspective of J. Dewey and G. H. Mead, but also on anthropologists T. Ingold and K. Milton’s current use of the affordance theory of J. Gibson and on G. Bateson’s theory of communication. The framework is elaborated systematically, along with a discussion about empirical studies of anthropomorphism, contemporary anthropological analysis of animism, and ethnographic studies of human–animal interactions. It tries to identify the conditions in which animal mental qualities can be perceived directly and it leads to a definition of anthropomorphism as the *situated direct perception* of animal minds (or other human properties) in the behavior or bodily expression of animals, by someone who is engaged in a specific process of activity. In a pragmatist perspective, anthropomorphism is a social activity that cannot be studied separately from its context of appearance. Its description and analysis must be achieved through careful ethnographic studies of real-life situations.

The first section examines previous studies of anthropomorphism and concludes that the concept of anthropomorphism as an act of inference is valid only in the context of the *scientific* activity of minding animals. In the non-professional activity of anthropomorphizing animals, people are not acting as distant or neutral observers of the animal’s behavior, but on the contrary, they are engaged in some kind of *dialog* with their environment. The *dialogic structure* of anthropomorphism (Airenti, 2012) will be discussed and analyzed in relation with G. Bateson’s theory of communication in the next section, leading to the conclusion that it is from the *inside* of a relationship (i.e., when they are affectively engaged) that people *see* human properties in animal’s behaviors or anatomical features. Thus, anthropomorphism does not only depend on characteristics that are present in the animal, but also on the kind of relationship and interaction between the person and the animal. In other words, the perceptual cues for the recognition of mental qualities are out there, in the animal, but they are discovered by someone who is *doing* something.

The fourth section will try to answer this question: if mental qualities of animals are directly perceived, can Gibson’s affordance theory (Gibson, 1979) account for anthropomorphism? In their analysis of animism in human–animal interactions, anthropologists Ingold (2000, 2002) and Milton (2002) convincingly claim that personhood is directly perceived by animist people in their environment. They provide ethnographic examples that clarify the kind of relationship in which such a perception occurs and call it a *relational epistemology*. In a relational epistemology, people turn their attention on what the animals (or plants) are *doing* to themselves and it seems that this affords for the direct perception of personhood in the surroundings. But the affordance theory is not enough for understanding of anthropomorphism because it leaves the social nature of the act of minding animals unexamined. The fifth section of the paper turns to empirical studies of human–animal interactions in the specific settings of the bio-medical laboratory. It is then obvious that the perception of affordances is not only learned, neither is it only dependent on the activity of the organism, it is also guided by materially and symbolically organized situations. To account for this, I introduce the concept of “perceptual frame.” A perceptual frame is both a definition of the situation in Goffman’s (1974) terms, and the performance of normative ways of attending to the animals, looking at them and letting oneself being affected – or not– by them. Through their very activity, people are trying to keep these perceptual frames alive and stable. Still, animals are living and acting beings, so unexpected affordances can emerge, inviting laboratory people to unexpected (or unwanted) kinds of relationships. In this perspective, animal’s (ontological and ethical) status is always provisional and unstable, and this is precisely what is found in the anthropological analysis of the (direct) perception of personhood in plants and animals, as well as in the ethnographic studies of human–animal interactions in laboratory facilities that are reported in the sixth section.

In contrast with the inference theory, the pragmatist perspective that I offer here is capable of considering the emergence of new significations in a situation. It shares the basic pragmatist view that perception is guided by the current activity: “What the sensation will be in particular at a given time, therefore, will depend entirely upon the way in which an activity is being used. It has no fixed quality of its own. The search for the stimulus is the search for exact conditions of action; that is, for the state of things which decides how a beginning coordination should be completed” (Dewey, 1896, p. 369). “Whatever we are doing determines the sort of a stimulus which will set free certain responses which are there ready for expression, and it is the attitude of action which determines for us what the stimulus will be” (Mead, 1936, p. 366). This perspective also assumes that people don’t “passively” and intellectually perceive the animals but that they rather “find” some stimulus in the animal’s behavior or anatomical structure that allow for the continued course of action. But the action is social, and in this, they are supported by culturally and socially learned modes of attention, ontological definitions of animals, and by materially and symbolically designed situations.

## Is Anthropomorphism an Act of Inference?

Many authors subscribe to a definition of anthropomorphism akin to the one provided by Guthrie (1997): anthropomorphism is “the attribution of human characteristics to non-human things or events” (p. 51). Even if definitions may differ on “what” precisely is attributed to the “non-human things and events,” there seems to be a consensus on the fact that anthropomorphism is a specific case of inference of something human to a non-human entity (Fisher, 1991; Eddy et al., 1993; Herzog and Galvin, 1997; Mitchell and Hamm, 1997; Silverman, 1997). In this perspective, anthropomorphism rests upon a cognitive work of inference.

This approach to anthropomorphism is indeed in line with the first theory of anthropomorphism, developed by Romanes and Morgan at the end of the 19th-century (Costall, 1993; Morris et al., 2000). Morgan proposed the word “ejective inference” while Romanes talked about “double inference,” in order to describe the double process of inference that supposedly took place when the scientist attributed mental qualities to an animal. The inference goes first from the observation of the animal’s behavior to one’s experience, where it is compared with one’s mental experience, and then it goes from one’s experience to the mental qualities attributed to the animal. According to these 19th century authors, inference of this kind was the only way in which a scientist could safely attribute intentions or other mental states to another animal. Because mental traits are “hidden” behind the behavior, they must be recovered through a double act of inference.

Yet, it should be noted that this kind of inference is the achievement of scientists who were searching for a safe way of studying animal minds in a Darwinian perspective. It is by no means a description of what people do when they are interacting with animals in everyday life. This has been made very clear by Costall (2007), who distinguishes between:

(1) Anthropomorphism as relating to other animals as subjects and agents, with feelings intentions, needs, and so on. This is what happens in everyday life, and it does not necessarily entail that we are dealing with “hidden” mental traits that are inferred;
(2) The *method* of anthropomorphism, a method committed to a dualism of mind and behavior. “This method assumes that making sense of animals as subjects necessarily entails an intellectual process of inference or “attribution” to bridge the gap between what we can observe (behavior) and what is supposed to be hidden (the mind), and such inferences are to be based on analogy from one’s own introspection” (Costall, 2007, p. 87).

Applying the model of anthropomorphism-as-inference as a general model of how people make sense of animals would mean considering the communicative and interactional structures that prevail in the behaviouristic operationalism of the laboratory as also operating in the life world. However, this is probably not the case. Indeed, the life-world is excluded by the practical methodology of the laboratory (Wieder, 1980; Rollin, 1990).

Researchers themselves have long noticed that, while they painstakingly try to use logical criteria to identify mental attributes in animals, their practical-minded assistants intuitively “find” mental phenomena that work for them (i.e., Silverman, 1997). Animal keepers rely on a very different (and much more efficient) way of understanding animals, which makes them able to *see* chimpanzees as conscious being or “embodied consciousness” (Wieder, 1980).

So, in spite of the fact that several authors insist that the researcher and the animal keeper (or the non-professional) are doing different things when they are minding animals, surprisingly, when it comes to empirically study anthropomorphism, the scientific stance (anthropomorphism-as-inference) is taken for granted and chosen as the model. Anthropomorphism is thus considered as a detached, decontextualized and intellectual operation, “one of many examples of induction whereby people reason about an unknown stimulus based on a better-known representation of a related stimulus, in this case reasoning about a non-human agent based on representations of the self or humans” (Epley et al., 2008, p. 145). Anthropomorphism is seen as a special case of the theory of mind, where “interpersonal understanding is seen as a theoretical accomplishment, involving a person constructing and using a “theory” of other people’s minds, as well as their own. Applying the theory to observable behavior enables the individual to interpret that behavior in intentional terms and as the product of specific mental states” (Leudar et al., 2004, p. 572).

It would nevertheless be obvious to a pragmatist that the activity of the detached and neutral observer (the scientist) and the activity of the non-professional who is affectively engaged in an interaction are two very different kinds of situations, which afford different ways of knowing. Disregarding this simple fact lead to the erroneous assumptions that similarity is a crucial determinant in anthropomorphism.

## Anthropomorphism and Human–Animal Similarity

As long as it is defined as the (decontextualized) attribution of human qualities to animals, anthropomorphism can be empirically studied through questionnaires asking people to attribute more or less complex cognitive and emotional states to animals. The first empirical studies of anthropomorphism (Eddy et al., 1993; Gallup et al., 1997; Herzog and Galvin, 1997) used this method and asked subjects to rate different animal species according to their supposed cognitive and emotional abilities. The results were rather convergent. They showed that the more the animals were considered similar or close to human beings, the more they were endowed with mental complexity. These results allowed Gallup et al. (1997, p. 91, my emphasis) to conclude that “the *use of anthropomorphism* appears to be influenced by the perceived similarity between humans and animals and the extent to which people have developed an affectionate bond with members of the species in questions (e.g., dogs and cats).” Additionally, the authors take these results as an evidence that anthropomorphism is, indeed, the result of an inferential work: “We contend that anthropomorphism is a by-product of self-awareness and the corresponding ability to infer the experience of other humans by using one’s own experience as a model” (p. 91). In opposition to this, I would state that what has actually been studied there is a cultural conception of animals that is only distantly related to anthropomorphism as it works in real-life situations. Actually, the results show what people commonly *think* about human–animal proximity and animal mental states – and, as Airenti (2012) reminds us, there is a big difference between believing that the coffee machine has intentions and behaving as if it had. Given the general education level of psychology students (who are often taken as subjects) the fact that they rate mammals as closer to human beings than invertebrate, and that they attribute more complex cognitive abilities to dolphins and apes than to pigs or rats is not surprising. Yet, knowing how people classify animals according to what they believe about their mental properties doesn’t say anything about what they do when they are actually interacting with them or even observing animals’ actual behaviors.

A study by Mitchell and Hamm (1997) specifies the role of perceived similarity in anthropomorphism. They gave undergraduates narratives depicting mammals’ behaviors (including human beings) suggestive of jealousy or deception. They then asked the subjects to evaluate their degree of agreement or disagreement with psychological characterizations of the animals described. The narratives presented various contexts and species (more or less close or familiar to human beings), but the behaviors remained constant. In these conditions, only variations *in the context* influenced the psychological characterizations. The species did not. The authors concluded that the main criteria for the psychological characterization of animals is the perceived structure of the “behavior-in-context.” This is not only more in accordance with the observations of Wieder (1980); Morris et al. (2000), and Servais (2012), it is also in agreement with the well-known fact that one can virtually attribute human properties to any object (Airenti, 2012). In Airenti’s examples, a piece of wood can become a “baby” in children’s play, and a coffee machine can be threatened by an angry user. Given this, we may doubt that similarity, or even plausibility, are the fundamental criteria for anthropomorphism. It might be the case when answering a questionnaire, but outside this very specific situation, something else is at play.

To make sense of seemingly contradictory experimental results of this kind, as regards anthropomorphism in children, Airenti (2012) suggested that anthropomorphism has two founding properties. Firstly, anthropomorphism is the expression of a basic teleological thinking, a way of *representing* non-human beings through their assimilation with human beings. Secondly, and most importantly, anthropomorphism manifests itself mainly in *interactions*. According to Airenti, for anthropomorphism to happen, it is necessary that the human characteristics be perceived in a specific interactional setting that she identifies as a *dialogic* relationship. She then suggests anthropomorphism should be seen as placing an object or an animal in the position of interlocutor in a dialogic relationship^1^ (Airenti, 2012, p. 49, my translation).

## The Dialogic Structure of Anthropomorphism

The implications of the dialogic structure of anthropomorphism for the perception of animal behavior may be examined along with G. Bateson’s theory of communication. In a paper about mammalian communication (Bateson, 1963), he suggests that every message (intentional or not) should be considered a two-sided entity: it is both a *report* and a *command*. It is a report about a past event (i.e., an emotion) and a command or a stimulus for a reaction of the partner (i.e., a threat). Or, in Bateson’s own terms: “The wag of the dog’s tail which for individual psychology signifies an inner state of the dog becomes something more than this when we ask about the functions of this signal in the relationship between the dog and his master. [……] It becomes an affirmation or a proposal about what shall be the contingencies in that relationship” (Bateson, 1963, p. 230). Simply speaking, the report is about the content of the message, while the command is about what the message does to the receiver, how it affects them and how it shapes the relationship. Every message has both aspects. Only the emphasis changes.

We can now see that the detached spectator (the scientist) is someone who makes oneself blind to the “command” aspect of a message. It means that they are not *affected* by the animal’s communicative signals. The signal is just a “report,” a bit of information about something else. Indeed, the best way to achieve neutrality when dealing with a living being, is precisely to make oneself impervious to the “command” aspect of the organism’s behavior or communicative signals. It is the safest way not to feel the urge to act when seeing, for example, a “depressed chimpanzee” (lowered body, slower pace, loss of appetite, increased response time…). The main point here is that precisely because the detached spectators keep themselves from being affected, *they won’t even see* a “depressed” chimpanzee; but only some behavior to be scientifically interpreted (i.e., neurophysiological cause). This deduction is in accordance with the phenomenological point of view that the perception of the behavior of certain things and beings is immediately given to us. Still this is only true for the *involved* consciousness, for “if we choose the ‘being-in-the-world’ of the detached spectator” this given understanding disappears (Buytendijk, 1952, p. 19, my translation).

For example, in one biology laboratory studied by Arluke (1988), rats about to be guillotined were kept in a separate room so that they could not see or smell the beheadings. This was justified on the grounds that “significant emotive changes in the rats produced by high-frequency distress calls would compromise the data.” (Arluke, 1988, p. 103). This is a good example of distress calls that are recognized as distress calls but do not call forth empathic responses as a distress call in a human infant might.

In the interactional setting of the disinterested or disengaged observer, inference is the only way to know about the animals’ mind. On the contrary, in a dialogic structure, because I agree to be sensitive and be affected by it, the animal’s experience becomes manifest through its expressive actions and body movements. Phenomenologists would say that knowledge of the animal’s mind is given through the contextualized apperception of its expressive body. “A crucial part of learning to be a “chimper” [namely a talented animal keeper] is learning to read chimpanzees body movements and gestures, that is, to see them as appresenting – to see, for example, arousal and anxiety in the slight erection of hair on the shoulders and in a particular bobbing motion *in some particular context*” (Wieder, 1980, p. 94, underlined by the author). Accordingly, in their paper arguing for animals as psychological beings, Bateson (1979) claim that expression is the heuristic route to direct knowledge of the mental states of others and that expression *is only visible from within* relationships (Bateson, 1979, p. 175).

Phenomenologically, a dialogic relationship can be conceptualized as a double move (Buytendijk, 1952). There is a move toward others in order to seize them (and this is the first property of anthropomorphism identified by Airenti) – and there is a move of offering, giving ourselves up in such a way that something might happen to us. Such a move can only be found when one agrees to be receptive to the “command” aspect of animal’s behavior, signals, or even anatomical shape or color. In the case of the piece of wood that becomes a baby, cited above, the child is responsible for the piece of wood’s moves, but nonetheless sees them as expressive movements, and responds accordingly. Inside this creative “as if” relationship, and only from the inside of this relationship, is the child able to *see* the piece of wood as a baby. For anyone else, it is just a piece of wood^2^.

We are now in a position to conclude that mental qualities are directly perceived from the inside of a relationship. How can this be, if nothing is inferred? For phenomenologists like Buytendijk or Wieder, mental states are directly appresented by expressive bodies: we do not meet bodies, but embodied consciousness. Could a pragmatist framework shed some light on the very question of the direct perception of mental – or human – properties? If we use Gibson’s affordances theory, I think it could. This isn’t aberrant. Both approaches have much in common, even if they differ on some points (Noble, 1981). Moreover, this theory has precisely been used by anthropologists who sought to analyze animism – which is the perception of human qualities in the natural environment.

## Affordances and the Direct Perception of the Environment

Gibson’s theory of affordances (Gibson, 1979) is the theory of a direct perception of the environment by a subject who is involved in his environment. It has been used by anthropologists Ingold (2000, 2002) and Milton (2002) to conceptualize the relationship between people and their natural environment, including animals. Affordances are “properties of the real environment as directly perceived by an agent *in a context of practical action*” (Ingold, 2002, p. 46, my emphasis). Affordance theory postulates that information is present in the environment, it doesn’t need to be constructed by a subject. Meaning is not imposed, nor “attributed” by a disengaged observer upon environment, but it is *discovered* by someone who is implicated in, and oriented by a practical action. “The man throwing the stone did not, we suppose, first “construct” the stone as a missile by attaching a meaning or “throw-quality” to impressions of it received through the senses. […]. Rather, it was the very involvement of the man in his environment, in the practical context of throwing, that led him to attend to the “throwability” of the object, by virtue of which it was perceived as a missile. Such direct perception of the environment is a mode of engagement with the world, not a mode of construction of it” (Ingold, 2002, p. 44).^3^ In its insistence on the discovering of properties in the environment according to the involvement of the subject in a practical action, Gibson’s perspective sounds very much like pragmatism. Perception is guided by the practical action, and the environment exist *for* a given organism. Indeed, organism and environment make “an inseparable pair” (Gibson, 1979, p. 18).

When she tries to understand the complex relationships that English conservationists have developed with the nature they strive to protect, anthropologist Kay Milton also draws on Gibson’s theory of direct perception. This is particularly so when she addresses the question of the “personification” of nature (Milton, 2002, pp. 42sq). Consistently with Ingold, she makes it clear that environmentalists don’t make nature and natural things *into* persons, they don’t *construct* them as persons. Rather, they *see* them as persons: they “discover the personhood of nature and natural things by perceiving their person-like affordances” (Milton, 2002, p. 45).

It is important to note that the perception of “person-like” affordances in animals or natural things does not happen in *any* kind of relationship or interactive situation. Many people live among animals and don’t see them as persons. In her search for the interactive conditions of the personification of animals, Milton turns to the work of Bird-David, an anthropologist who studied the Nayaka hunter-gatherers of South India. These people have a specific way of relating themselves to their environment that Bird-David called a “responsive relatedness” (Bird-David, 1999). Responsive relatedness is a way of engaging one’s attention to the surroundings. The Nayaka are attentive to the changes of things in the world *in relation to themselves*. In other words, their attention is turned to what things in their environment *do* to themselves rather than what they are. “Animals and other objects which actively engage their attention, stones which ‘come toward’ or ‘jump on’ them, elephants which ‘walk harmlessly’ or ‘look straight into the eyes’ are perceived as having a kind of personhood” (Milton, 2002, p. 46).^4^ Milton adds that the sort of environmental knowledge the Nayaka express, and which Bird-David called a “relational epistemology,” has been identified many times by anthropologists, particularly in hunter-gatherer cultures. Many North American hunters describe not only animals, but a wide range of other natural phenomena as “persons” including trees, rocks, winds, the sky, and so on. This has generally been understood as evidence that hunter gatherers “believe” that animals, plants, wind, etc. have psychological properties and intentions. Nevertheless, Milton notes that such an interpretation is a gross falsification that led to a deep misunderstanding of animism. It is due to our modernist point of view, which sees animism as the attribution of personhood *to* natural things (through inference) rather than the perception of personhood *in* these things. In relational epistemology, personhood is not a property of something, it emerges out of what something does in relation to others. Ingold (2000) shares Milton’s analysis. For him, when Cree hunters describe their reindeer prey as *offering* its life to the hunter, they are not making a statement of fact about the reindeers. Rather, their description should be understood as “a performance of which aim is to give form of human feelings” (Ingold, 2000, p. 25) where feeling is “a mode of active, perceptual engagement, a way of being literally ‘in touch’ with the world” (ibid, p. 23). In other words, the Cree’s description is mistaken by the modernist observer as being about the “report” aspect of communication, although it should be understood as an account of the “command.” In the perspective opened by Ingold, the Cree’s description of the hunt will not be misunderstood as a “weird” or irrational conception of animals anymore. On the contrary, it is a very accurate and precise description of the experience of the hunter of being touched and moved by his prey.

The cultural interpretation varies, of course, but relational epistemology is probably not restricted to hunter-gatherer societies (Bird-David, 1999; Milton, 2002). I would argue that in both societies, our sensitivity to the personhood of non-human animals depends on the intensity with which they engage our attention and respond to what we do.

This discussion shows that the perception of personhood *in* the environment happens when people are sensitive to animals *in relation* to themselves. More precisely, they are sensitive to their own response to the animal’s behavior or anatomical features. In this situation, one doesn’t “construct” nor infer mental properties, but feels or sees them. In Bateson’s language of relations, we would say that the animal’s body or behavior affords a certain kind of relationship and that the mental qualities that are perceived “in” the animal emerge from this felt relationship. In this relational perspective, there is an interesting rapprochement to be noted with G. H. Mead’s reflexion about how objects acquire their “interior.” According to Mead, an object “gets its inside when it arouses in the organism its own response and thus the answering response of the organism to this resistance” (Mead, 1959, p. 136?).^5^

The theory of affordances allows us to understand how it is that a perceptual salience born by an animal (i.e., anatomical structure, a behavior, a gesture, or any specific shape) will be discovered or not by a human being, according to the practical action in which they are engaged. Now, the problem of anthropomorphism may be phrased as follows: how does it come to be that certain “traits” or “structures” on the animal (or plant) are “selected” and “aggregated” instead of some (or no) others? The affordance theory suggests that the kind of practical action in which one is engaged is determinant in the perception of affordances. However, the theory alone doesn’t help when it comes to the description and analysis of these practical actions and how they frame and constrain perception. Moreover, as Noble (1981) has perceptively noted, the theory itself is unable to account for the *social meaning* with which some objects are endowed. Noble claims that Mead’s theory of the social object is able to solve some of the problems encountered by Gibson when it comes to social meaning. As the next section will clearly show, animals are attended to in social settings. There is a *framing work* that organizes the perception and the attention of the people engaged in corresponding actions. As I conceptualize it, this framing work is realized both symbolically (through language and many other symbolic acts) as well as materially (through material devices such as chains, cages, etc.). As I see it, such perceptual frames are enacted permanently by people through their coordinated actions and perceptions in a situation. Still, because animals are living beings that do unexpected things, these perceptual frames are challenged and fragile I have chosen “perceptual frame” over Meads theory of the social act because the latter cannot account for the instability of emergent significations in the situation, nor can it help to single out specific frames as objects for investigation.

## Keeping the Pig in the Right Perceptual Frame

In his late work “Frame Analysis” (Goffman, 1974), the sociologist E. Goffman used the concept of *frame* to refer to the (mostly implicit) social definition of a situation. Each situation needs to be defined or framed as a specific occurrence of something, for example, “interacting with a pet dog.” According to the situational definition, some perceptual (behavioral, anatomical, etc.) cues will be perceived as affordances for the current action. In a pragmatist view, there is a mutual definition of the perceptual frame and the practical action. As the perceptual frame helps guiding the action and discovering the affordances for the action, the current action confirms and stabilizes the perceptual frame so that the practical action can continue.

Coming back to laboratory life, it is clear now that between the scientists who make themselves blind to the “command” aspect of communication, and do not attribute mental qualities to animals, and the animal caretakers who engage in a subject-to-subject relationship with the same animal, and perceive it as minded, the difference is not just in the act of inference. It is not that they perceive the same animal but differ in their willingness to infer mental qualities. Rather, I argue that they *perceive* (or *enact*) different animals because they are engaged in different actions with them, within different interaction regimes. The extensive ethnographic work of Arluke (1988) in biomedical laboratories and their animal facilities offers many examples that provide a better understanding how technical, symbolic and practical devices contribute to construct and stabilize perceptual frames in the life world of their face-to-face interactions with animals.

Arluke’s (1988) main finding is that laboratory animals don’t have a single status but, on the contrary, are seen as objects *and* pets. He documents the transformation of “naturalistic” animals^6^ into either objects or pets as a “social construction” of the laboratory animals. Here my focus will stay on the practical interactive conditions in which each status is actualized and how it affects anthropomorphism. From the point of view of pragmatism, what is constructed is less the animal itself than the perceptual frame in which the animal is directly perceived *as* object or pet. The “construction” work happens upstream from the face-to-face human–animal interaction. For example, animals are objectified through a set of procedures that involve technical, material and symbolic devices (cages, codes, etc.) that deprive them of their individuality and expressive capacities. These procedures and devices define the current activity and ascribe it to a recognizable category of activities. But their function is also to prepare laboratory workers to *perceive* animals *as* – mainly objects. They organize the activity toward the animals and orient perception. They help laboratory caretakers, technicians and scientists avoid being sensitive to the “stimulus” aspect of the animal’s behavior. When these procedures fail, laboratory workers resort to specific strategies that help them keep the animal in the right perceptual frame. Arluke precisely describes the de-anthropomorphizing strategies used to objectify animals. Interestingly, they mainly have to do with perception and can be seen, in a broad sense, as “education of attention” (Ingold, 2001) devices.

(1) Animals are de-individualized, treated as a collective entity and labeled with a code that refers to the experiment in which they are enrolled. De-individualization not only facilitates the redefinition of the animal’s nature. It also materially prevents laboratory workers from *seeing* them as individuals. In a laboratory, one post-doctoral student was asked to stop naming the sheep because it made it harder for the others to conduct their experiments. I would say that naming changes the perceptual frame: when they have a name, animals have the power of making themselves present in the eye of the human being. Their behavior and expressive movements now afford for a subject-to-subject relationship and this challenges their objectification.
(2) Animal bodies are deprived of expressive capacities. There is a strict separation between the experimental and the care-taking spaces and people try to avoid having conscious animals in the laboratory. When it couldn’t be avoided, dog cages were kept facing the wall, and a surgical sheet was draped over the cages. Scientists usually don’t see the animals while they are conscious. Yet, when it happens accidentally, the situation may be completely reframed, as it is the case in this example: “one day, [the P.I.] came into the laboratory when a dog was still awake, tied by a rope leash to the surgical table. He looked at the dog, mumbled, ‘oh, god, what will my wife say now!’ turned around, and left” (Arluke, 1988, p. 104). In another example, three technicians and two post-doc fellows had to wait for the P.I. while three conscious dogs were waiting for anesthesia in the laboratory. Absolutely no attention was given to the dogs, even when someone had to pass the dogs, and even when the dogs then approached the human, wagged their tails and tried to make eye contact. “There was no acknowledgment that the dogs were present” (Arluke, 1988, p. 105).
(3) Situational definition. According to Arluke, “nothing in the animal itself solely determines this definition” (Arluke, 1988, p. 104). Indeed, in one laboratory, one of the guinea pigs was selected randomly by the technicians as the laboratory mascot and pet. It was given a human name and was particularly admired for its intelligence. It was taught tricks and technicians found its behavior to be endearing. When it broke its leg in a cage accident, it underwent surgery to fix it. Next door, a dog similarly broke its leg but was consequently killed. This example shows clearly that it isn’t some inherent properties of the animals that will trigger anthropomorphism or mental states attributions, but rather the perceptual frame that allows for the perception of some behaviors or properties as affording engagement and social interaction. Affordances may be present on the animal, but it is the course of action and interaction that determines which ones are perceived, and for what.

The final example is about failing to keep the animals (pigs) in the right perceptual frames. In an experiment, pigs had to be attended 24 h a day by technicians (who became known as pig-sitters). Their job was to sit at a desk, two feet away from the pen in which pigs were kept, to monitor the technical equipment, to record the pigs’ global activity and to keep the pen clean. Three months later, the pigs were sacrificed for additional data. In these circumstances the technicians couldn’t avoid developing strong attachment to the pigs. The pigs were named after super-heroes and the pig-sitters were sincerely fond of them. Although they tried not to develop a pet relationship with the pigs, it was impossible for the pig-sitters to see them simply as laboratory objects. Because of the intricacies of their respective lives, the pigs and their pig-sitters shared a story, they were in a dialogic relationship and the pig-sitter were affected by the pigs. With the growing familiarity, the pig-sitter’s perception of their animal charge became more acute; the pig became present not as an experimental body, but as an embodied consciousness. Sacrifice, Arluke writes, “was clearly a collective trauma” (Arluke, 1988, p. 115).

## Fragile Perceptual Frames?

As the previous examples have shown, the animal’s status varies enormously depending on the practical actions the human beings make them parts of. Herzog (1988) documented the case of “escaped” mice in a laboratory. The escaped mice once lived as experimental subject, but they managed to escape and since then, they live an underground life and changed status: now they are bad mice that need to be exterminated. According to Herzog, the label “good” or “bad” mouse explains why individuals of the same species receive such different treatments: while the good ones are killed with kindness, the others are cruelly trapped. In agreement with Noble (1981), I don’t think that the name of a thing in and of itself causes it to be perceived in one way or another. Rather, mice change status because people act differently toward them; as escaped mice, they offer different bodily and behavioral cues, and they engage people in different actions (trap, destroy…) that in turn cause them to behave differently. For the pragmatist, who considers action prior to perception, the name is second: they become bad ones because they are cruelly trapped.

The observations of Fluvian (2010) may provide some additional understanding. She too has observed that mice are given several statuses (living being, preparation and sensitive being), but no name is attached to it. Interestingly, she has noted that when the mouse status changed, *the whole interactive situation changed*: the researcher’s tone of voice while talking to the mouse, her facial expression, the way she handled and perceived the mouse. Again, it would be difficult to argue that the perception of the mice’ mental qualities depends on an act of inference that would proceed cognitively from behavioral cues and analogical reasoning in one situation but not in another one. Objectively, it can be argued that the cues are probably present in each situation, but the practical conditions of the action and interactive settings are making them obvious (affordances) or invisible. When shifting from a detached to an engaged position, the researcher perceives or enacts another mouse.

Actually, it is well known in anthropological research that animal status can change abruptly, in a rapid process that challenges the whole definition of the situation. In many hunter-gatherer societies, ontological differences between human beings and animals are far from fixed. They are rather “chronically unstable” and require efforts (i.e., relational processes) to be both stabilized and transformed (Remme, 2016, p. 118). Even in our society, in the most “fixed” perceptual frame, as is the laboratory, it may happen that a simple “look” on an animal’s face unexpectedly challenges the course of action. One laboratory studied by Arluke decided to call off one of the experiments because laboratory technicians were convinced that the dog to be sacrificed “knew what was happening” because of “something in his eyes and behavior.” In one way or another, that dog managed to make his personhood perceptible in spite of the objectifying perceptual frame. It can happen that an unexpected affordance arises from a perception that is peripheral with regard to the main action and the main definition of the situation. Then, an alternative signification takes shape and the whole situation is reframed. In this case, sacrifice became murder, and the action became impossible to carry out. This is why laboratory people develop strategies to keep these competing affordances in the background of their awareness. It should be emphasized that this is a never-ending process. Cultural devices help stabilize the status of animals, but these are always provisional.

In the pragmatist view advocated here, perceptual cues, like a dog offering itself for petting, work as social affordances which invite particular kinds of behavior, and not others. According to the current action, they will be perceived or not. In any event, the interaction is the context in which mental states are perceived. It can even be argued that the perception of affordances, like the dog inviting me to stroke her, is directly linked to the apperception of mental states. As I perceive the dog’s invitation to stroke her, I feel her as friendly. Maybe I’ll later verbalize it as “she is kind,” but it is not necessary, as I can stay in the feeling of being related to this “kind” animal. Additionally, it is misleading, as this verbalization is only a *post hoc* utterance that pretends to describe the dog while it is indeed about my feeling of the dog and my relationship with her.

## Anthropomorphism and Imagination

Before I conclude, I’d like briefly re-examine the question of imagination in anthropomorphism. According to the pediatrician and psychoanalyst Donald Winnicott, imagination is necessary to relate oneself to something that is different from one’s self. He created the concept of “intermediate area” or “potential space” to name “an intermediate area of experiencing, to which inner reality and external life both contribute” (Winnicott, 1971, 2005, p. 3). This intermediary area that could support an encounter with something very different from one’s own self contains the possibility of establishing a relation with the world that does not force the individuals to choose between the inner life and the outer reality, but, quite on the contrary, enables them to connect the inside and the outside in a creative way. The example of the girl playing with a piece of wood as a baby is a good example of an experience taking place in an intermediate area. I would hypothesize that, maybe in many animal encounters, the creation of an intermediate area is the condition for people to be able to aggregate their experience and, thanks to imagination, connect the heterogeneous perceptual cues afforded by the animal’s body and/or behavior, and recognize some pattern.

While the example of the girl reminds us of the potential role of imagination in anthropomorphism, the concept of intermediate area cautions us against a radical view of anthropomorphism as a pure projection of human properties onto animals. In the scope of this theory, anthropomorphism is better defined as a way to perceive/create patterns that connect people with animals and make them relevant according to the current activity. This perspective is radical in the sense that we no longer need to decide whether some features (i.e., jealousy), “really” belong to the animal behavior *or* are projected by the human observer, but instead it invites the researcher to empirically document the cultural, interactive and situational conditions in which it happens.

## Conclusion

This paper has shown that anthropomorphism, when it is studied in its naturally occurring circumstances, appears to be more complex than the attribution of mental or human qualities to an object, event or living being, according to a similarity gradient. As many examples have shown, anthropomorphism is not so much the product of an act of inference as it is the direct perception of human properties by someone who is engaged in a specific interaction and who accepts to let him/herself being touched or affected by the animal and its expressive qualities. Personhood is perceived rather than attributed, and it is perceived by the whole body, not only by the mind. Because the human or mental qualities are perceived from the inside of a relationship, keeping a relational point of view on anthropomorphic terms would prevent confusing them with a description of the animal “itself” while they are truly about the human–animal relationship.

From a pragmatist point of view, if it is true that animal mental qualities are discovered/produced in a specific interactional setting, it follows that any description of animal mental qualities should be accompanied by a description of its relational context of discovery. This could also be the case for the scientific inquiry in animal minds, as it has been suggested that animals are differently minded according to the interactional regime. Actually, this kind of reflexive thinking is usual in anthropology and, from a pragmatist point of view, could have its rationale in cognitive ethology too. Finally, the paper also suggests that uncertainty, imagination and illusion could be considered as important ingredients of human–animal relationships. Considered the situated perception of human and/or mental qualities, anthropomorphism appears as a powerful lens through which human–animal relationships can be studied. The perspective that has been advocated here also offers conceptual tools for in-depth ethnographic studies of anthropomorphism as a complex situated phenomenon.

## Author Contributions

The author confirms being the sole contributor of this work and has approved it for publication.

## Conflict of Interest Statement

The author declares that the research was conducted in the absence of any commercial or financial relationships that could be construed as a potential conflict of interest.

## Footnotes

1 Freely translated by the author from Airenti (2012, p. 49): “Toute familiarité est donc liée à la possibilité de placer un objet inanimé dans la position d’interlocuteur dans un dialog.”

2 This example should not be taken as a claim that minding animals from the inside of a relationship equates to hallucinating non-existent movements. Still, it reminds us that anthropomorphism is more or less has linked with affect and imagination.

3 Note that Tim Ingold has turned to affordance theory to object to the traditional anthropological conception of animals as “cultural construct” – a very old anthropological view that assumes that the entire signification is in head of the human being and that nature, animals, plants, etc. are pure materiality.

4 We could extend the example that Ingold has given about the stone and the perception of its “throw-quality” affordance by saying that in the kind of attention that is characteristic of responsive relatedness, I can even hear the stone saying “take me.”

5 Examining this convergence more thoroughly in the scope of further research would be of interest.

6 This is what Lynch (1988) called the “biological” animal in the laboratory. He opposed it to the “analytical” animal, which is a source of reliable scientific information. In a Science and Technology Studies approach, Lynch’s analysis aims at describing the process by which the analytical animal is constructed.
